# Possible solution for coronary cannulation through complete anti-anatomical deployment of self-expandable transcatheter heart valve using handmade spiral curve of catheter created by heat-gun

**DOI:** 10.1093/ehjcr/ytaf011

**Published:** 2025-03-18

**Authors:** Masanori Yamamoto, Hirooki Higami, Yuki Kondo, Fubuki Kitahara

**Affiliations:** Department of Cardiology, Gifu Heart Center, 1-14-4, Yabutaminami, Gifu City, Gifu 500-8384, Japan; Department of Cardiology, Toyohashi Heart Center, 21-1 Gobutori, Oyamachyo, Toyohashi, Aichi 441-8530, Japan; Department of Cardiology, Nagoya Heart Center, 1-14 Sunadabashi, Higashiku, Nagoya, Aichi 461-0045, Japan; Department of Cardiology, Gifu Heart Center, 1-14-4, Yabutaminami, Gifu City, Gifu 500-8384, Japan; Department of Clinical Engineer, Nagoya Heart Center, 1-14 Sunadabashi, Higashiku, Nagoya, Aichi 461-0045, Japan; Department of Clinical Engineer, Nagoya Heart Center, 1-14 Sunadabashi, Higashiku, Nagoya, Aichi 461-0045, Japan

## Case description

**Figure 1:**
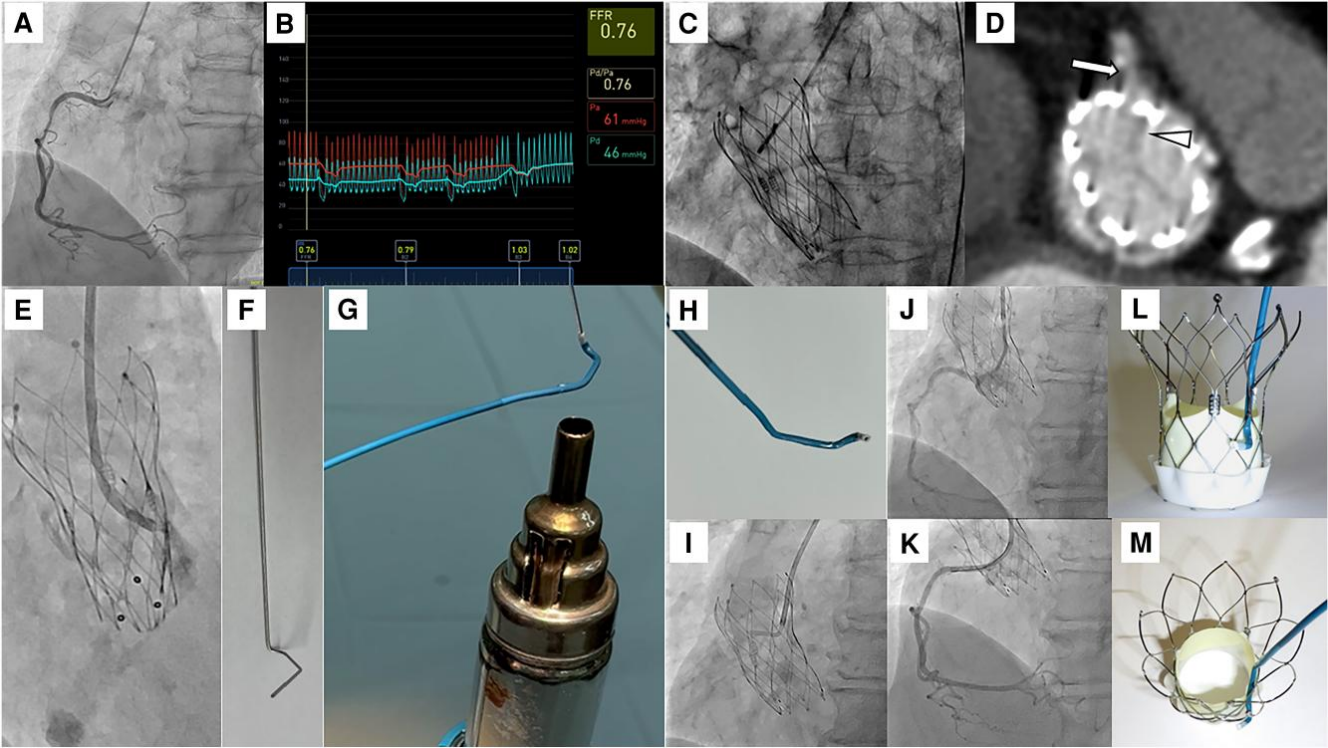
(*A*) Coronary artery angiography (CAG) revealed 75% stenosis in the middle and distal segment of right coronary artery (RCA). (*B*) The fractional flow reserve on invasive measurement was 0.76 at the distal part of RCA. (*C*) A successful transcatheter aortic valve replacement (TAVR) using intra-annular self-expandable transcatheter heart valve (SE-THV) was performed. (*D*) The post-procedural multi-detector computed tomography identified the complete anti-anatomical commissure position of SE-THV (white arrowhead) towards the RCA ostium (white arrow). (*E*) Despite multiple attempts of contrast injection near the RCA, there was poor visualization of RCA because of difficult selective engagement of 6 French (6Fr) guide catheter (GC). (*F*) The pre-shaped metallic rod was prepared to make a right spiral specific curve of GC. This metal rod was created to assume a shape that would allow the catheter to pass through the THV frame in a circular arc in a gyrating manner. The metal shape is formed and sterilized through pre-procedural simulation and is used prior to treatment. (*G*) After insertion of the straight 5Fr GC into the pre-shaped rod, the 5Fr GC could be re-shaped by manufactured heat gun within a temperature range of 150–200°C. To ensure optimal results while preventing potential damage to the GC, it is recommended that the GC correction process be performed in a few short seconds while maintaining around 5 cm between the heat gun and the GC. The metal rod and GC are then cooled with cold water. Finally, the metal rod is carefully removed from the GC and the shape of the GC is checked. (*H*) The whole image of handmade 5Fr spiral curve GC inside the 6Fr GC. When inserting this specific form of GC, there is concern about deformation if a 5Fr handmade catheter is inserted later into a 6Fr catheter. For this reason, it is preferable to insert the 5Fr catheter into the 6Fr catheter first before performing the deformation of the 5Fr catheter. With this process of preparation, the entire system can be guided to the coronary artery entry site supporting with a standard 0.035 soft wire. (*I*) The guide wire crossing and engagement of GC for RCA were able to utilize this specific inner GC system. (*J*) CAG showed no significant stenosis after coronary stenting. (*K*) The image represents the usefulness of spiral curve to navigate the GC closing to the RCA. This spiral curve was well designed to avoid the interference of commissure post of SE-THV.

An 83-year-old woman diagnosed with severe aortic stenosis and coronary artery disease (CAD) was admitted to our hospital. Coronary angiography confirmed 75% stenosis in the right coronary artery (RCA) with evidence of significant myocardial ischaemia (*Figure 1A* and *B*). As the CAD was stable, the patient initially underwent transcatheter aortic valve replacement (TAVR) with a Navitor-VISION 25 mm (Abbott, Santa Clara, CA, USA) intra-annular self-expandable transcatheter heart valve (SE-THV) (*Figure 1C*). After successful TAVR, elective percutaneous coronary intervention (PCI) for the RCA was planned. Based on post-procedural multi-detector computed tomography, the commissure post of the SE-THV was placed in front of the RCA ostium (*Figure 1D*). Although a 6 French (Fr) Judkins Right 4.0 guide catheter (GC) was used to engage the RCA, adequate engagement and selective injection were difficult (*Figure 1E*). The floating technique of a 0.014-inch guide wire (GW) supporting the microcatheter was also ineffective because the commissure post of the SE-THV prevented the penetration of the GW towards the ostium of the RCA. After several failed attempts of using conventional methods, we created a handmade right spiral curve of a 5Fr UNITE (Asahi Intecc, Aichi, Seto, Japan) catheter using a specific iron core and a manufactured heat gun (*Figure 1F* and *G* and movie). Combined the 6Fr GC with a handmade 5Fr catheter system (*Figure 1H* and *I*), the 0.014-inch GW could cross the lesion, and the GC was adequately inserted into the RCA supporting the inner 5Fr catheter (*Figure 1J*). With coaxiality to the RCA ensured by the insertion of the GW, the inner 5Fr catheter could be removed and the 6Fr GC alone was used for treatment. Finally, the two coronary stents were successfully implanted (*Figure 1K*). The schema of the handmade spiral curve GC provided a possible solution for engaging the RCA (*Figure 1L* and M). One of the reasons for unsuccessful PCI through the THV was the anti-anatomical position of the SE-THV towards the coronary ostium. Whether PCI should be performed before or after TAVI depends on the patient's background and severity of CAD. If PCI after TAVI is chosen, the physicians should be aware of commissure alignment during the TAVI procedure. The patient had difficulty successfully aligning the THV, and discussion of THV selection and technical improvements should continue. The created spiral shape of the GC was considered an ideal curve to overcome the difficulties encountered during PCI after SE-THV.

## Supplementary Material

ytaf011_Supplementary_Data

## Data Availability

The data underlying this article are available in the article and its online Supplementary material.

## Notes

**Consent:** Informed consent was obtained from the patient for the publication of this case report and accompanying images.

**Funding:** None declared.

